# 642. The Role of Electronic Medical Record Automation in Latent Tuberculosis Screening and Treatment in a Large Health System

**DOI:** 10.1093/ofid/ofae631.207

**Published:** 2025-01-29

**Authors:** Hannah T Li, Harleen Sahni, Andrea Cervenka

**Affiliations:** Santa Clara Valley Medical Center, San Jose, CA; Santa Clara Valley Medical Center, San Jose, CA; Santa Clara Valley Medical Center, San Jose, CA

## Abstract

**Background:**

Valley Medical Center is a tertiary county hospital in San Jose, California that also staffs 12 clinics. Our institution’s Mycobacterial and Refugee Health Clinic is the main referral center for latent tuberculosis (LTBI) and active TB cases in the county.

The USPFTF recommends screening for LTBI in asymptomatic adults who are at higher risk. This is challenging in primary care settings where physicians often see a high number of patients within limited time frames. There are many points on the LTBI care cascade (Figure 1) where patients can fall off. In the age of automation, technological tools can be designed to help reduce clinician burden.

**Figure 1:**
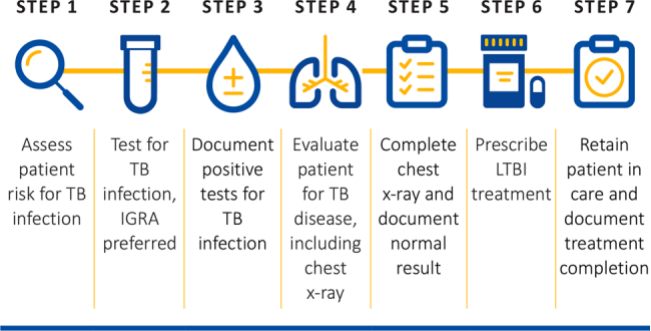
Latent Tuberculosis Care Cascade Source: California Department of Public Health Tuberculosis Control Branch

**Methods:**

An EMR tool was engineered to prompt physicians to order a QuantiFERON (QFN) if a patient meets certain criteria: 18 years or older, no prior QFN or TB skin test, and any of the following risk factors: HIV positive, in custody, drug use, homelessness, or non-English primary language (proxy for foreign birth). This EMR tool went live on 8/9/22.

Using EMR data-collection tools, a retrospective analysis was done among patients who had a clinical encounter (office visit or telemedicine) between 8/9/21-8/9/22 and 8/9/22-8/9/23 (one year before and after implementation of the tool). We determined the number of patients who were eligible for screening, obtained a QFN, had a positive QFN, initiated, and completed treatment. A 2-sample Z-test was used to assess temporal trends.

**Table 1:**
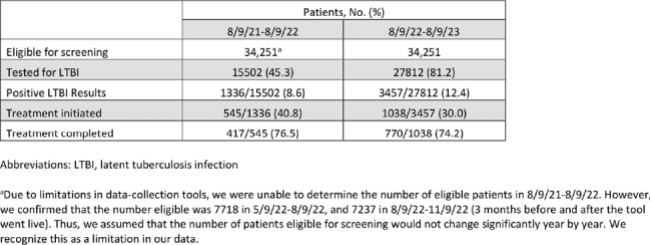
LTBI Care Cascade for Patients Seen by a Primary Care Physician in 8/9/21-8/9/22 and 8/9/22-8/9/23

**Results:**

From 8/9/22-8/9/23, 34251 patients were marked eligible for LTBI screening based on the EMR tool (Table 1, Figure 2). The proportion of patients tested for LTBI increased from 45.3% to 81.2% (p-value < 2.2e-16). The proportion of positive LTBI results increased from 8.6% to 12.4% (p-value < 2.2e-16). The number of patients who initiated treatment increased from 545 (40.8%) to 1038 (30%). The number of patients who completed treatment increased from 417 (76.5%) to 770 (74.2%).

**Figure 2:**
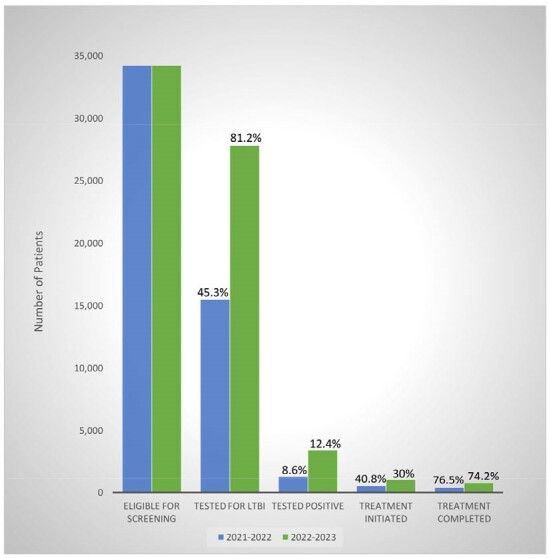
LTBI Care Cascade for Patients Seen by a Primary Care Physician in 8/9/21-8/9/22 and 8/9/22-8/9/23

**Conclusion:**

This study highlights that a low-cost EMR intervention can lead to substantial increases in LTBI screening, and as a result higher treatment completion. EMR automation can also improve accuracy in screening, reflected by the increased proportion of positive QFN results. Future tools involving digital technology can be designed to reduce drop-offs at other points along the LTBI cascade as well.

**Disclosures:**

**All Authors**: No reported disclosures

